# MEK inhibition, alone or in combination with BRAF inhibition, impairs multiple functions of isolated normal human lymphocytes and dendritic cells

**DOI:** 10.1186/2051-1426-1-S1-P93

**Published:** 2013-11-07

**Authors:** Laura J Vella, Anupama Pasam, Nektaria Dimopoulos, Miles Andrews, Anne-Laure Puaux, Jamila Louahed, Ashley Knights, Weisan Chan, Katherine Woods, Jonathan Cebon

**Affiliations:** 1 The Ludwig Institute for Cancer Research, Melbourne, VIC, Australia; 2 GlaxoSmithKline, Rixensart, Belgium; 3 La Trobe University, Melbourne, VIC, Australia

## Introduction

Combination therapy with BRAF and MEK inhibition is currently in clinical development for the treatment of BRAF mutated malignant melanoma. BRAF inhibitors are associated with enhanced antigen-specific T-lymphocyte recognition in vivo. Consequently BRAF inhibition has been proposed as pro-immunogenic and there has been considerable enthusiasm for combining BRAF inhibition with immunotherapy. MEK inhibitors inhibit ERK phosphorylation regardless of BRAF mutational status and have been reported to impair T-lymphocyte and dendritic cell function. In this study we investigate the effects on isolated T-lymphocytes and monocyte-derived dendritic cells (moDCs) of a MEK and BRAF inhibitor combination currently being evaluated in a randomized controlled clinical trial.

## Experimental design

The effects of a BRAF inhibitor and a MEK inhibitor, alone and in combination were studied on isolated normal T-lymphocyte and moDCs. Lymphocyte viability, together with functional assays including proliferation, cytokine production and antigen-specific expansion, were assessed. moDC phenotype in response to lipo-polysaccharide stimulation was evaluated by flow-cytometry, as were effects on antigen crosspresentation.

## Results

BRAF inhibition did not have an impact on T-lymphocytes or moDCs, whereas MEK inhibition alone or in combination with BRAF inhibition suppressed T-lymphocyte proliferation, cytokine production and antigen-specific expansion. Similarly, moDC cross-presentation was suppressed in association with enhanced maturation following combined inhibition of MEK and BRAF. However no significant decrease in CD4+ or CD8+ T-lymphocyte viability was observed following kinase inhibition.

## Conclusions

MEK inhibition, alone or in combination with BRAF inhibition can suppress immune cell function and further studies in vivo will be required to evaluate the potential clinical impact of these findings.

